# Immunotherapy-associated dysgraphia as an early neurocognitive manifestation of immune effector cell–associated neurotoxicity syndrome: clinical characteristics, mechanistic insights, and assessment challenges

**DOI:** 10.3389/fimmu.2026.1731798

**Published:** 2026-04-29

**Authors:** Yasuto Yamamoto, Togen Masauji, Takeo Shimasaki

**Affiliations:** 1 Department of Pharmacy, Kanazawa Medical University Hospital, Kahoku, Japan; 2 Medical Research Institute, Kanazawa Medical University, Kahoku, Japan

**Keywords:** CAR-T therapy, cognitive dysfunction, dysgraphia, ICANS, neurotoxicity, T-cell engager, writing impairment

## Abstract

**Background:**

Immune effector cell therapies, including chimeric antigen receptor T-cell (CAR-T) therapy and T-cell–engaging antibodies, are associated with immune effector cell–associated neurotoxicity syndrome (ICANS), a clinically recognized immune-mediated neurotoxicity. Among its neurological manifestations, writing impairment (dysgraphia), including paligraphia and other forms of writing disturbance, has emerged as a potentially sensitive clinical marker of higher-order cortical dysfunction.

**Objective:**

This review synthesizes current clinical evidence on ICANS-associated dysgraphia, characterizes its clinical and neurocognitive features, and examines limitations in existing assessment frameworks.

**Methods:**

A structured literature search was conducted using PubMed to identify reports describing writing impairment associated with immune effector cell therapies. Studies were selected through title and abstract screening, with additional relevant reports identified through manual reference screening to address limitations of keyword-based indexing.

**Results:**

The available evidence consists primarily of case reports and small series describing early-onset dysgraphia, frequently characterized by paligraphia, spatial disorganization, and executive dysfunction. Writing impairment typically emerges within days of infusion and often occurs during the early phase of ICANS. Although its precise frequency remains unknown, published reports suggest that dysgraphia may be under-recognized rather than truly rare. Importantly, these findings indicate that ICANS-associated dysgraphia represents a heterogeneous spectrum of writing abnormalities that is not adequately captured by current assessment tools such as the ICE score, which rely on binary evaluation.

**Conclusions:**

Dysgraphia represents an under-recognized but clinically meaningful manifestation of ICANS and may serve as an early and sensitive indicator of higher-order cortical dysfunction. The development of structured and quantitative handwriting assessment strategies may improve early detection and monitoring of neurotoxicity in patients receiving immunotherapy.

## Graphical Abstract

**Figure:**
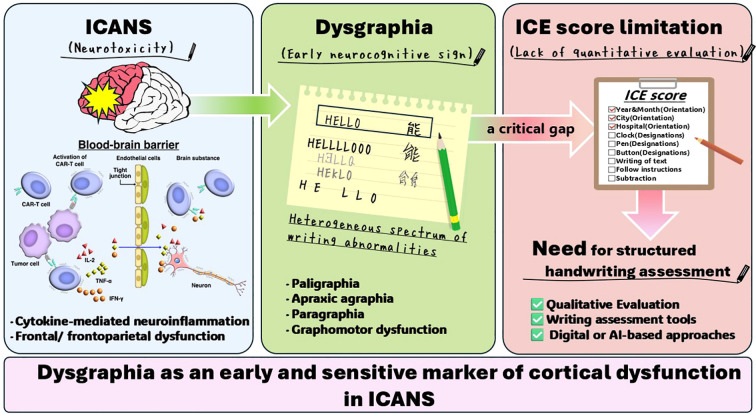
Immune effector cell therapies, including CAR-T cells and T-cell–engaging antibodies, can induce immune effector cell–associated neurotoxicity syndrome (ICANS). Cytokine-mediated neuroinflammation and blood–brain barrier disruption may lead to transient dysfunction of frontal cortical networks involved in executive and language processing. As a result, dysgraphia, including paligraphia and other writing abnormalities, may emerge as an early neurocognitive manifestation of ICANS. However, current assessment frameworks such as the ICE score rely on binary evaluation and do not capture qualitative handwriting abnormalities. Structured handwriting assessments, potentially supported by digital or AI-based approaches, may improve early detection and characterization of ICANS-related neurotoxicity.

## 1 Introduction

### 1.1 ICANS overview

Immune effector cell therapies, including chimeric antigen receptor (CAR) T-cell therapy and T-cell–engaging bispecific antibodies, have dramatically improved outcomes for patients with refractory hematologic malignancies. However, these therapies are also associated with immune effector cell–associated neurotoxicity syndrome (ICANS), a potentially serious neurological complication that can manifest as cognitive dysfunction, language disturbance, seizures, or encephalopathy. Because early neurological changes may be subtle, sensitive bedside indicators of cortical dysfunction are critically needed to facilitate early detection and monitoring of ICANS.

Among these potential indicators, writing impairment (dysgraphia) has recently attracted attention. Writing is a complex cognitive activity integrating language processing, executive control, visuospatial coordination, and fine motor planning. Consequently, disturbances in handwriting may reflect early dysfunction within distributed cortical networks. Several recent reports have suggested that dysgraphia may appear during the early phase of ICANS, sometimes preceding more overt neurological deterioration (1–4). Nevertheless, the clinical characteristics, pathophysiological implications, and optimal assessment strategies for ICANS-associated dysgraphia remain insufficiently synthesized in the literature.

**Figure 1:**
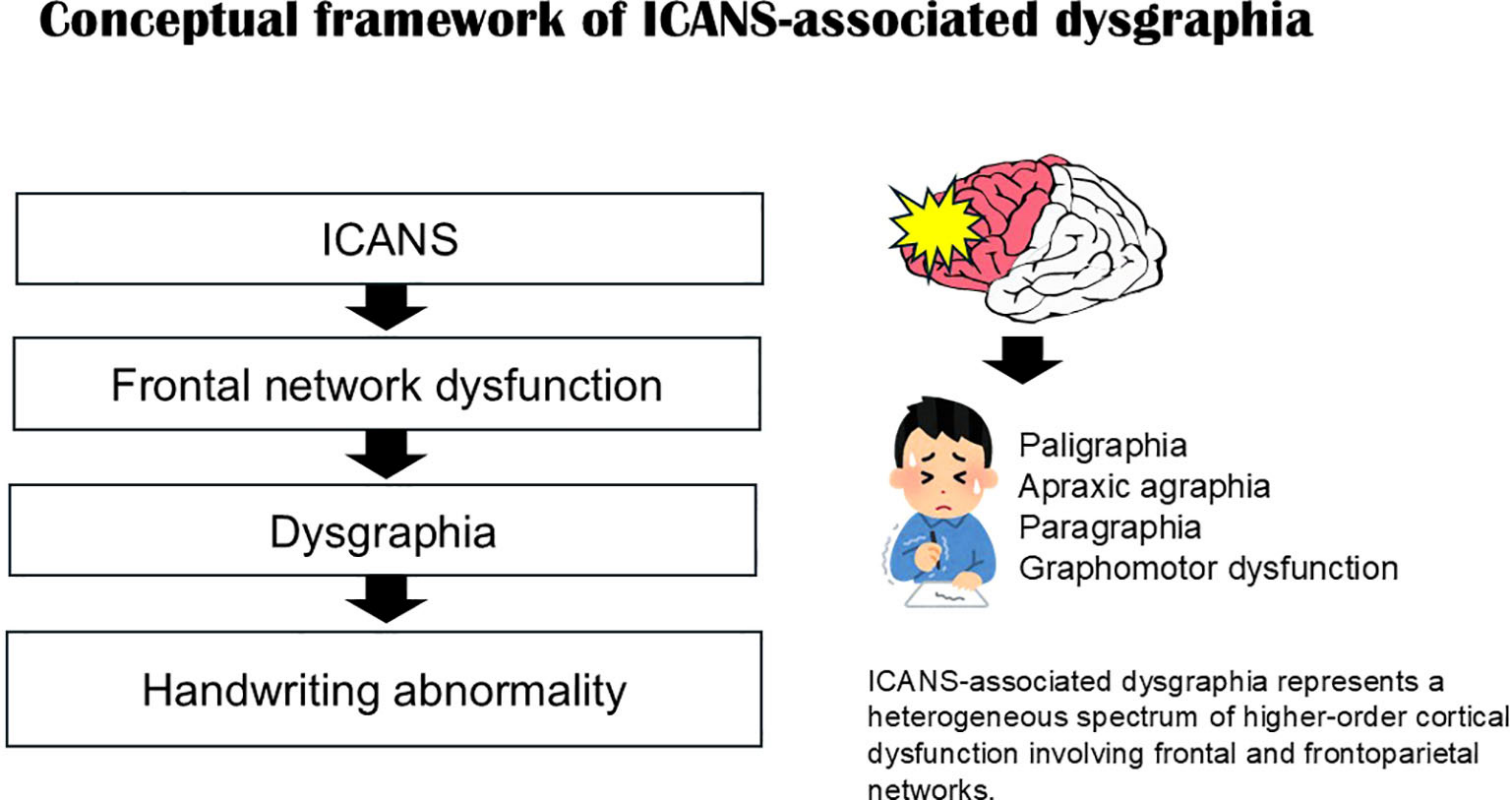
Conceptual framework of ICANS-associated dysgraphia.

Writing is a higher-order cortical function that depends on the integration of language, executive control, visuospatial processing, and fine motor execution (5–7). This conceptual relationship between ICANS, frontal network dysfunction, and dysgraphia is summarized in **Figure 1**.

Recent reports have described dysgraphia—including paligraphia (pathological repetition of letters or words)—as an early feature of ICANS. However, writing impairment is rarely analyzed in detail, and its frequency, characteristics, and assessment remain insufficiently synthesized.

This review aims to (1) summarize available clinical evidence on ICANS-associated dysgraphia (2), describe its characteristic features, including paligraphia and frontal involvement, and (3) evaluate limitations in current assessment frameworks.

## 2 Materials and methods

Literature search strategy and study selection:

A literature search was conducted using PubMed to identify studies reporting writing impairment associated with immune effector cell-related neurotoxicity. The search was performed in the title/abstract fields using the following query, and only articles published in English were considered.

(dysgraphia OR agraphia OR handwriting OR writing OR aphasia OR language disturbance OR language disturbances OR cognitive dysfunction OR cognitive dysfunctions)

AND

(CAR T OR CAR-T OR chimeric antigen receptor OR immune effector cell OR blinatumomab OR T-cell engager OR T-cell redirecting bispecific antibody)

AND

(ICANS OR neurotoxicity OR neurologic toxicity OR encephalopathy)

The initial search yielded 54 records; after title and abstract screening, 42 were excluded, and 12 studies were assessed in full text.

We recognized that writing impairment is often described indirectly within broader neurocognitive or frontal dysfunction and may not be explicitly indexed using writing-related terms in titles or abstracts. Therefore, to address this limitation, manual reference screening was performed. Through this process, one additional relevant study (8) was identified and included as a manually added reference.

This combined approach, integrating systematic database searching with manual reference screening, was adopted to minimize the risk of missing clinically relevant evidence due to indexing limitations.

Eligibility assessment and data extraction:

Full texts of potentially relevant articles were assessed for eligibility. For each included study, the following data were extracted: Type of immunotherapy; Description of writing-related symptoms; Neurotoxicity context (e.g., ICANS, encephalopathy); Evaluation framework or assessment tools used (e.g., ICE score).

Studies were categorized as explicit (directly reporting writing impairment) or implicit (writing impairment incorporated within neurotoxicity assessment frameworks).

**Figure 2:**
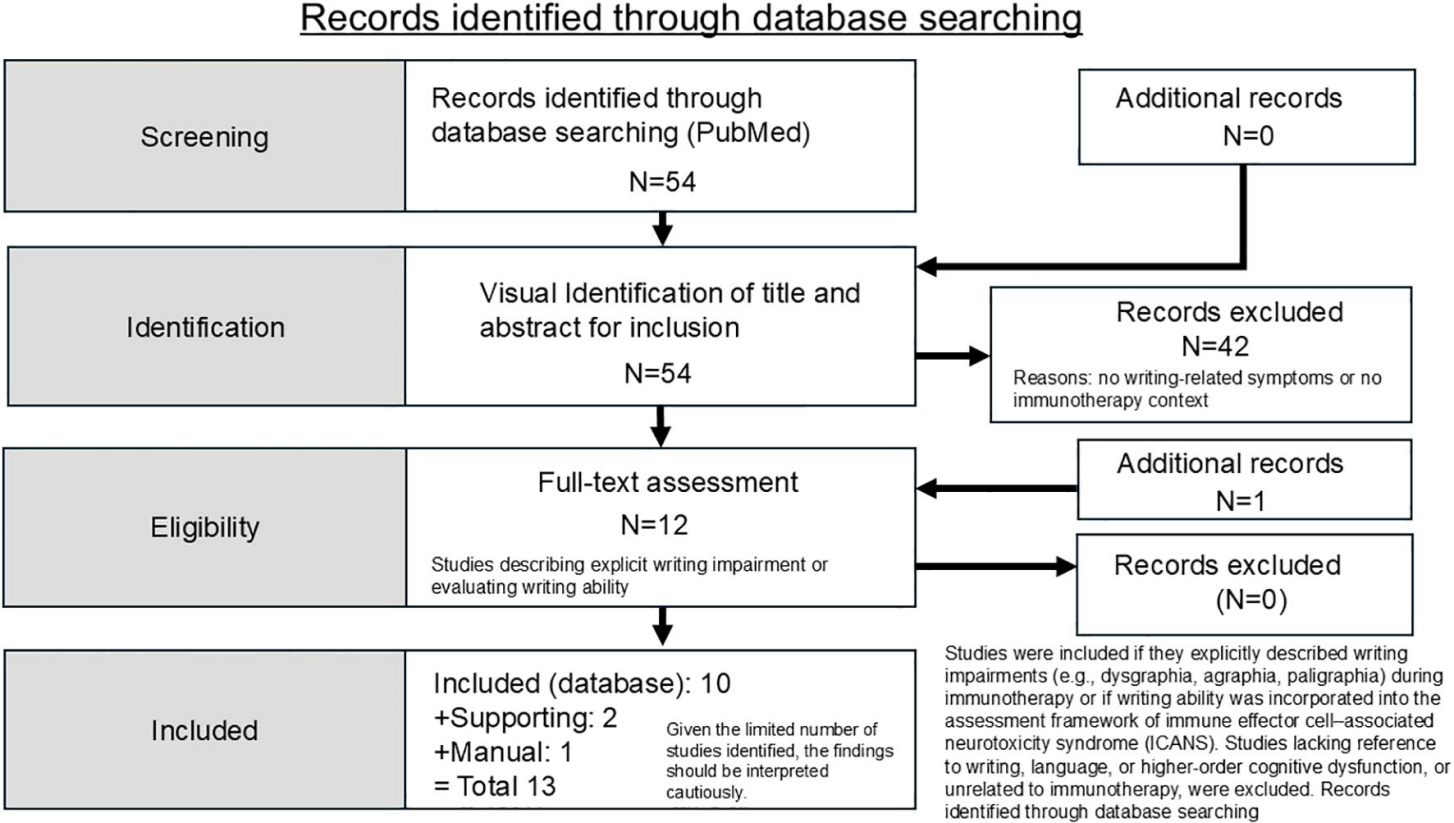
Literature search and selection flow diagram. Given the limited number of included studies and the reliance on case reports and small series, the findings should be interpreted with caution.

Study evaluation and synthesis:

Due to the heterogeneity of study designs and outcome measures, a qualitative synthesis was performed. The final set of included studies represents publications in which writing impairment was either a documented clinical manifestation or an inherent component of standardized neurotoxicity evaluation following immunotherapy. The literature identification and selection process is summarized in a flow diagram (**Figure 2**; **Supplementary Tables 1**, **2**). The literature search was initially conducted prior to the original submission and was subsequently updated during the revision process to ensure inclusion of recently published relevant studies.

Database selection:

Given the clinical and neurological focus of the topic, PubMed was selected as the primary database to ensure relevance and reproducibility.

## 3 Results

A total of 54 records were identified through database searching. After title and abstract screening, 42 records were excluded. Twelve studies were assessed in full text. Of these, 10 studies met the inclusion criteria and were included as database-identified studies. In addition, two studies were included as supporting references, and one study (Fontanelli et al. (8)) was added through manual reference screening. The literature identification process is summarized in **Figure 2**; **Supplementary Tables 1**, **2**.

### 3.1 Emergence of novel neurotoxicities with immune-based anticancer therapies

The 1998 International Council on Harmonization of Technical Requirements for Medicinal Products for Human Use guidelines recommend using the common definitions and assessment criteria to facilitate common understanding, assessment, and acceptance of clinical trial data from foreign countries. Currently, the Common Terminology Criteria for Adverse Events (CTCAE), version 5.0, developed by the National Cancer Institute, is widely used to evaluate adverse events in cancer therapy clinical trials (9). However, the CTCAE guidelines may not adequately capture or grade emerging types of adverse events associated with newer anticancer agents. One example is dysgraphia, which may appear during the onset of central nervous system (CNS) events attributed to immunotherapy with effector T-cell therapy or bispecific T-cell engager therapy (1, 3).

Effector T-cell therapies involve engineering patient-derived T cells to express chimeric antigen receptors (CARs) that recognize tumor-associated antigens. After reinfusion, CAR-expressing T cells directly recognize and eliminate malignant cells. In addition, T-cell–engaging bispecific antibodies redirect endogenous T cells to tumor targets. Available effector T-cell therapies include tisagenlecleucel, axicabtagene ciloleucel, idecabtagene vicleucel, and ciltacabtagene autoleucel. Other T-cell engager therapies expected to have antitumor effects by enhancing binding to T cells are being developed. The mechanism of action involves the transient cross-linking of the patient’s cytotoxic T cells and CD19- or CD20-positive malignant B cells through the antibody, which activates the T cells to act on the target B cells. Available T-cell engager therapies include blinatumomab, which targets CD19, and epcoritamab, which targets CD20.

### 3.2 Clinical characteristics of ICANS-associated dysgraphia

#### 3.2.1 Dysgraphia as an early manifestation of ICANS

**Figure 3:**
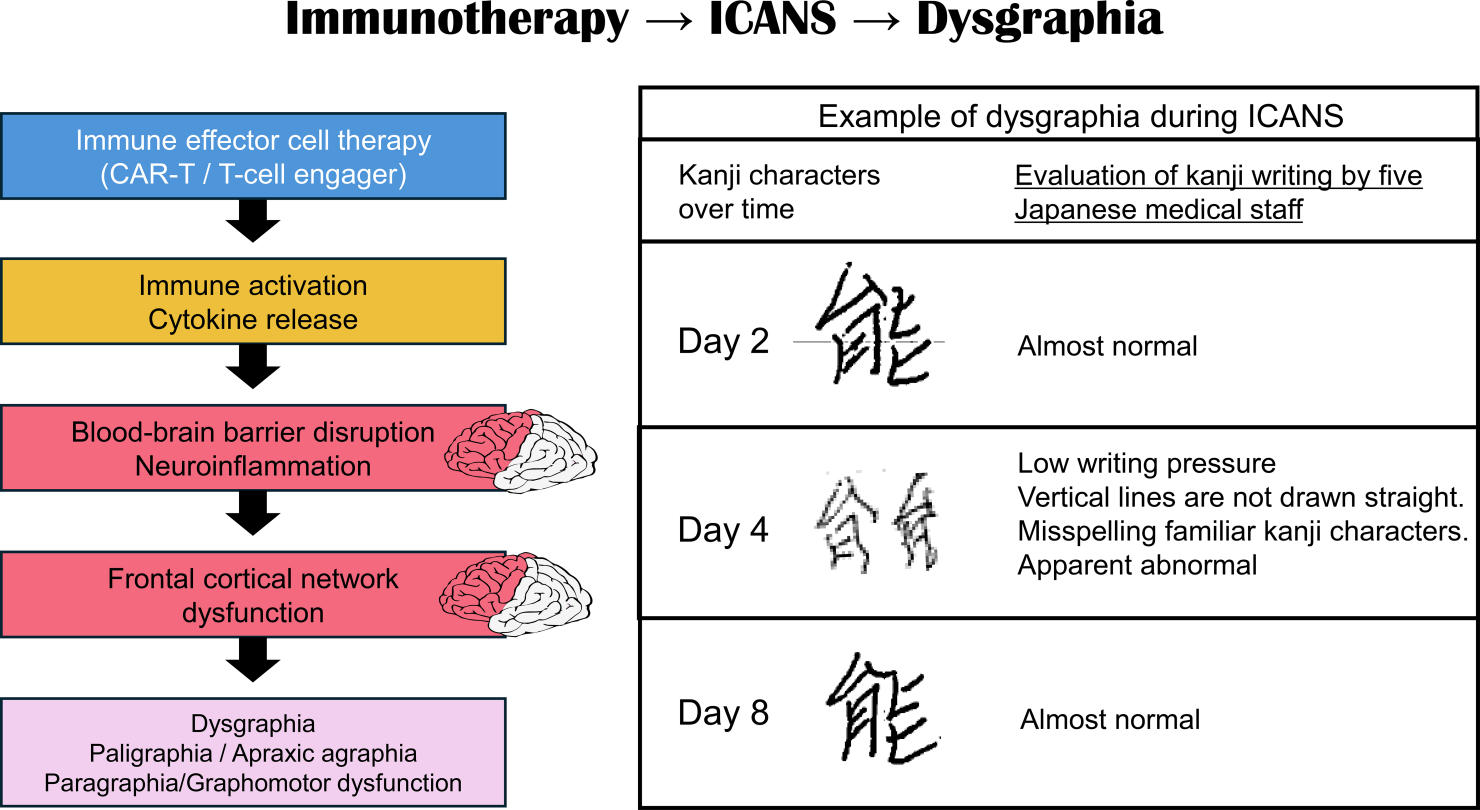
Immunotherapy-induced ICANS leading to dysgraphia. Immune effector cell therapies can induce cytokine-mediated neuroinflammation and blood–brain barrier disruption, leading to dysfunction of frontal cortical networks. This network disruption may manifest clinically as dysgraphia during ICANS. Representative handwriting changes are reproduced from our previous report (Ref. 1).

These immune cell therapies are associated with a clinically recognized neurotoxicity known as immune effector cell–associated neurotoxicity syndrome (ICANS). This condition has been characterized as a pathological process occurring in the CNS resulting from the activation and targeting of intrinsic or extrinsic T cells or other immune effector cells. Symptoms and signs may be progressive and include aphasia, impaired consciousness, cognitive impairment, muscle weakness, convulsions, and cerebral edema. However, no clear cause has yet been established. ICANS typically develops within the first week after immune effector cell infusion. Depending on the therapeutic product and cohort, ICANS incidence ranges from approximately 20% to 60% (9–13). Published case reports indicate that writing impairment often emerges early in the course of ICANS (1–3), sometimes preceding more overt neurological deterioration. Dysgraphia has been described as reversible and temporally associated with peak inflammatory activity. Because writing requires intact executive and language networks, early dysgraphia may reflect frontal network dysfunction even when global consciousness remains preserved. The temporal evolution of these handwriting abnormalities during ICANS is illustrated in **Figure 3**.

Currently, the management of chemotherapy side effects is evaluated using CTCAE based on patient complaints and laboratory findings. However, CTCAE alone has been proven inadequate for ICANS specific to immunotherapy, prompting the development of the immune effector cell-associated encephalopathy (ICE) grading system (9) along with the level of consciousness, presence of seizures, and increased intracranial pressure. Although these efforts have facilitated the assessment of ICANS severity, other problems remain.

#### 3.2.2 Paligraphia and frontal lobe involvement

**Figure 4:**
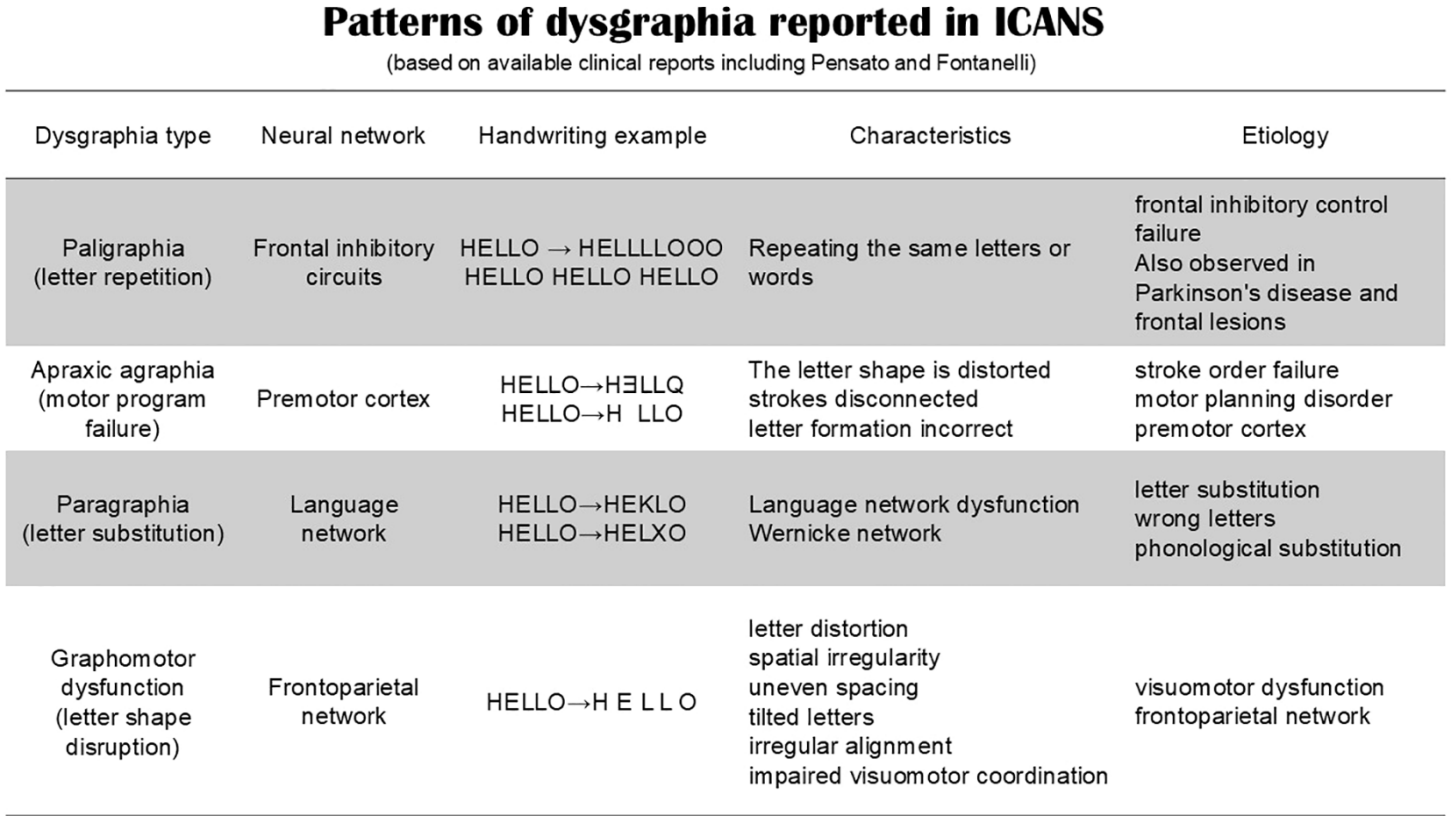
Patterns of dysgraphia reported in ICANS (based on available clinical reports, including Pensato et al. and Fontanelli et al.). Writing disturbances include paligraphia, apraxic agraphia, paragraphia, and graphomotor dysfunction, reflecting impairment of distributed cortical networks involved in executive control, language processing, and motor planning.

Paligraphia has been described in several ICANS cases; however, writing disturbances associated with ICANS appear to be heterogeneous. For example, Fontanelli et al. (8) reported a case series in which writing disturbances consisted of a combination of apraxic agraphia, paragraphia, and paligraphia, while paligraphia itself was absent in one of the patients. Pensato et al. (2, 3) reported frontal-predominant encephalopathy associated with early paligraphia following CAR-T therapy. The reported patients developed writing abnormalities within days of infusion, accompanied by executive dysfunction and imaging findings consistent with frontal involvement. The spectrum of dysgraphia patterns reported in ICANS is summarized in **Figure 4**.

Fontanelli et al. (8) further described writing disturbances in CAR-T recipients, reinforcing the association between ICANS and higher-order cortical dysfunction. These reports suggest that ICANS-related dysgraphia may reflect transient dysfunction of frontal-subcortical networks rather than peripheral motor impairment. The presence of paligraphia supports involvement of executive and inhibitory control systems, functions primarily mediated by the frontal lobes.

These findings collectively indicate that paligraphia represents only one component of ICANS-associated dysgraphia and should be interpreted within a broader spectrum of writing disturbances involving multiple cortical networks.

### 3.3 Drug-specific observations

Writing impairment has been described across multiple immune effector cell therapies:

- Axicabtagene ciloleucel: Axicabtagene ciloleucel has been associated with early ICANS presenting with language disturbances, including aphasia and encephalopathy, and case-based evidence has described writing abnormalities such as paligraphia-like features (2, 14).
- Tisagenlecleucel: Tisagenlecleucel has also been associated with neurotoxicity in clinical trials, including encephalopathy and language dysfunction, although detailed descriptions of writing abnormalities remain limited (13, 15).
- Blinatumomab: Blinatumomab has been associated with neurotoxicity, and case reports have documented handwriting distortion and difficulty writing complex characters during treatment (1, 16).
- Epcoritamab: Epcoritamab has been associated with ICANS and language-related neurological manifestations, including aphasia, based on clinical trial data, although detailed descriptions of writing abnormalities remain limited (17, 18).

In most reports, dysgraphia developed within several days after infusion and resolved with improvement of neurotoxicity. However, detailed documentation of dose-response relationships and standardized characterization remains limited.

### 3.4 Estimated frequency and reporting limitations

Although ICANS incidence is reported in major trials, specific reporting of isolated dysgraphia is lacking. Neurotoxicity is typically categorized globally (e.g., “encephalopathy,” “aphasia,” “confusion”) without granular documentation of writing abnormalities. The limited number of published dysgraphia cases likely reflects under-recognition and absence of structured assessment protocols rather than absence of the phenomenon (19–23). Previous reviews have similarly suggested that subtle neurocognitive changes, including handwriting abnormalities, may be under-recognized in routine clinical practice (24). No trial to date has systematically quantified qualitative features of writing impairment.

### 3.5 Pathophysiological considerations

**Figure 5:**
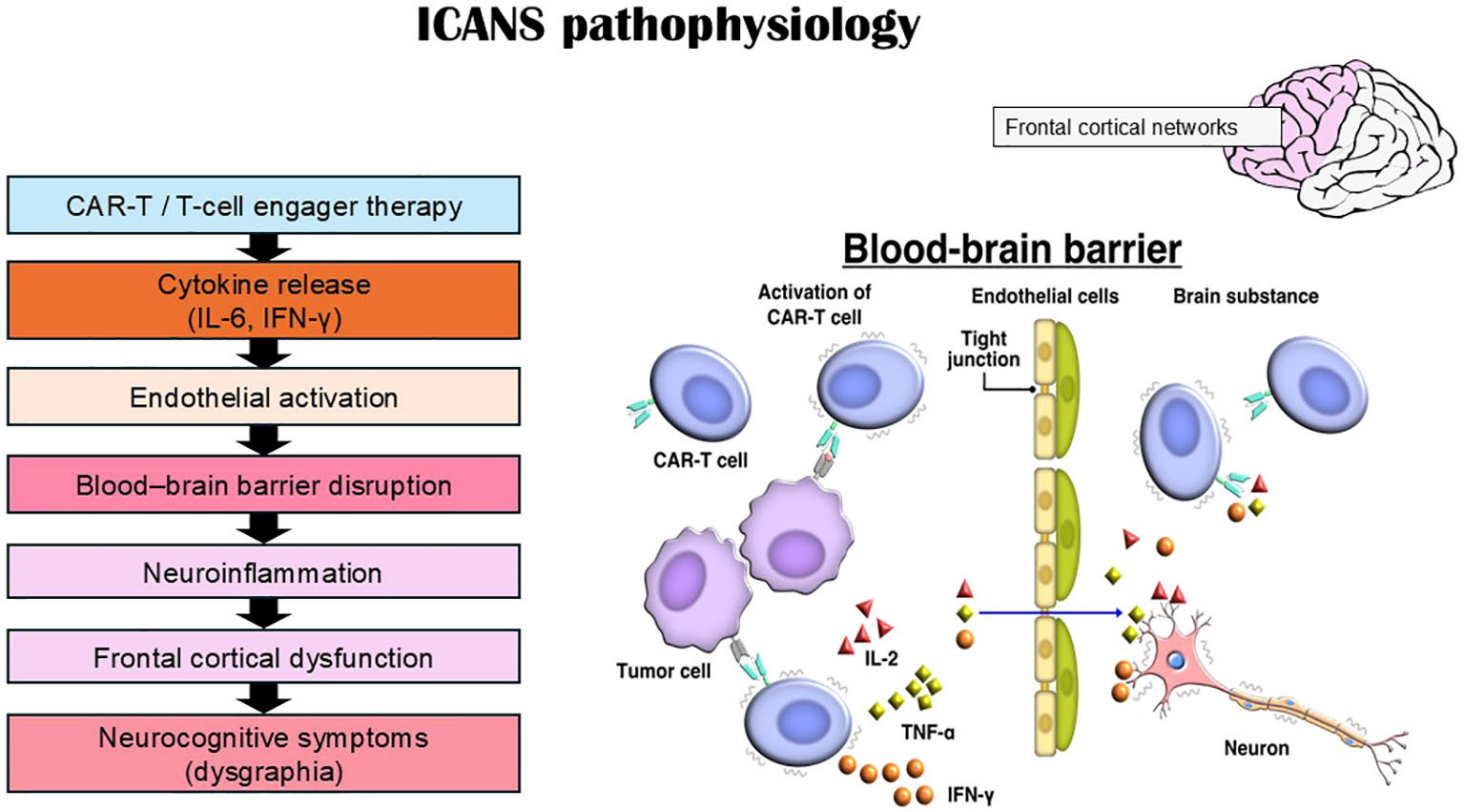
Proposed pathophysiological mechanisms of ICANS-associated dysgraphia. Cytokine release and endothelial activation following immune effector cell therapy may disrupt the blood–brain barrier and trigger neuroinflammation. Frontal cortical networks involved in executive and language functions may be particularly vulnerable, resulting in dysgraphia and other cognitive disturbances.

The proposed pathophysiological cascade underlying ICANS-associated dysgraphia is summarized in **Figure 5**. The precise mechanisms underlying ICANS remain incompletely elucidated. Proposed mechanisms include (25–28):

- Cytokine-mediated endothelial activation
- Blood–brain barrier disruption
- Neuroinflammation
- Microvascular dysfunction

Emerging mechanistic reviews further support the role of cytokine-mediated neuroinflammation and network-level dysfunction in ICANS-associated neurotoxicity (29). Activated immune cells and elevated cytokine levels may disrupt cortical networks, particularly in frontal regions, leading to executive dysfunction and language impairment. Endothelial activation and blood–brain barrier disruption have also been identified as key contributors to ICANS-related neurotoxicity (30). The transient nature of many ICANS cases suggests reversible functional network disruption rather than structural neuronal injury. Frontal vulnerability may partially explain the early emergence of paligraphia and executive-related writing disturbances.

### 3.6 Limitations of current assessment frameworks

#### 3.6.1 ICE score

The Immune Effector Cell–Associated Encephalopathy (ICE) score includes a sentence-writing task within its 10 point assessment. The evaluator instructs the patient to write a sentence and directly evaluates the written output. However, scoring is binary and does not quantify qualitative distortions (9) such as repetition, spatial disorganization, or letter complexity–dependent impairment.

**Figure 6:**
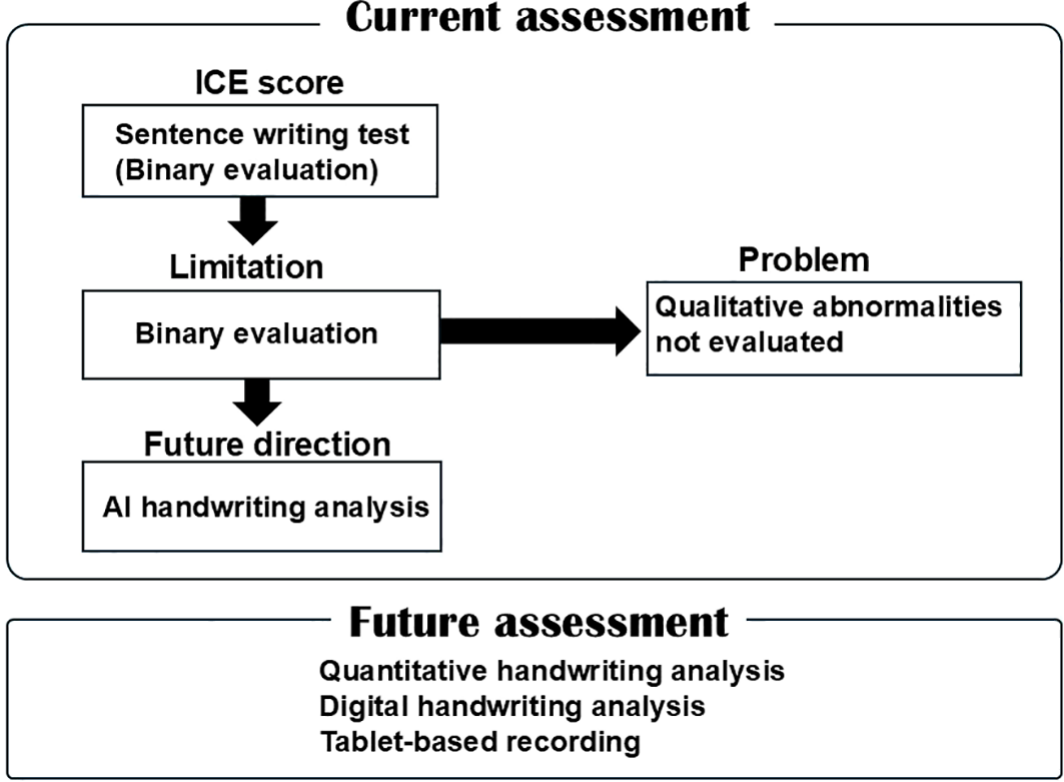
Limitations of current handwriting assessment in ICANS monitoring. The ICE score includes a sentence-writing task in which the patient is required to produce a written sentence; however, evaluation is primarily binary and does not capture qualitative handwriting abnormalities.

Subtle writing abnormalities may therefore be missed, particularly when patients remain able to produce a syntactically valid sentence. In addition, structured handwriting assessments in clinical settings have demonstrated that subtle writing abnormalities may be detectable even when global cognitive scores remain relatively preserved (31). The structure of the ICE grading system is illustrated in **Figure 6**.

#### 3.6.2 CTCAE limitations

The Common Terminology Criteria for Adverse Events (CTCAE) does not include a dysgraphia-specific grading system. Writing impairment is indirectly referenced under broader language or cognitive categories but lacks structured severity assessment.

As a result, there is currently no standardized grading framework for dysgraphia in the context of ICANS.

### 3.7 Clinical characteristics and neurocognitive mechanisms of ICANS-associated dysgraphia

Writing is a complex cognitive function that requires the integration of multiple neural systems, including language processing, visuospatial organization, executive control, and fine motor planning. Because of this complexity, disturbances of writing can arise from dysfunction at several levels of the cortical network. In the context of immune effector cell–associated neurotoxicity syndrome (ICANS), writing abnormalities have increasingly been recognized as an early clinical manifestation of cortical dysfunction.

Several recent reports have described writing disturbances in patients undergoing CAR T-cell therapy. Among these, Pensato et al. reported cases characterized by early paligraphia associated with frontal encephalopathy following CAR T-cell infusion (2). Paligraphia refers to the pathological repetition of letters or words and is generally considered a manifestation of impaired inhibitory control within frontal–subcortical circuits.

However, subsequent observations indicate that ICANS-associated dysgraphia is not limited to paligraphia. These heterogeneous patterns of dysgraphia are summarized in **Figure 4**. In a case series of sixteen patients treated with CAR T-cell therapy, Fontanelli et al. (8) reported writing disturbances in two patients who developed ICANS. These disturbances included a combination of apraxic agraphia, literal paragraphia, and paligraphia, suggesting impairment of multiple components of the writing network. Notably, one of the two patients exhibited apraxic agraphia and paragraphia without paligraphia, indicating that repetition phenomena are not a universal feature of ICANS-related dysgraphia.

Apraxic agraphia is thought to result from a disruption in the retrieval of motor engrams required to generate written letters, typically associated with premotor or frontal dysfunction (32). Literal paragraphia reflects the substitution or misplacement of letters within words and may involve language-related cortical regions. Paligraphia, in contrast, reflects perseverative behavior associated with impaired inhibitory control.

Taken together, these observations suggest that ICANS-associated dysgraphia should be considered a heterogeneous clinical phenomenon (2, 7, 8), potentially reflecting transient dysfunction of frontal and frontoparietal cortical networks rather than a single specific syndrome.

Emerging evidence further supports the hypothesis that ICANS frequently involves frontal lobe dysfunction. Both clinical observations and neurophysiological findings have demonstrated features consistent with frontal encephalopathy, including behavioral changes, executive dysfunction, and motor perseveration. In the study by Fontanelli et al. (8), both patients who developed ICANS exhibited subtle signs of frontal lobe impairment at baseline neurological examination, such as palmo-mental reflexes, motor impersistence, and reduced saccadic velocity. These findings raise the possibility that pre-existing frontal vulnerability may predispose certain patients to develop ICANS.

The mechanisms underlying this frontal predominance remain incompletely understood. One proposed explanation involves cytokine-mediated neuroinflammation leading to blood–brain barrier disruption and cortical network dysfunction. Experimental data suggest that frontal cortical regions may be particularly susceptible to inflammatory signaling pathways, including NF-κB-mediated responses. This vulnerability may contribute to the early emergence of executive and language-related symptoms such as dysgraphia during ICANS.

Given these findings, careful assessment of writing function may provide clinically valuable information during monitoring for ICANS. Current screening tools, including the Immune Effector Cell–Associated Encephalopathy (ICE) score, incorporate a simple sentence-writing task. However, this assessment primarily evaluates whether a sentence can be produced and does not capture qualitative abnormalities such as letter repetition, structural distortion, or motor planning deficits. More detailed characterization of writing disturbances may therefore improve early detection and phenotypic classification of ICANS-related neurotoxicity.

Future studies should aim to systematically document the qualitative characteristics of writing impairment in larger patient cohorts. Such investigations may help clarify whether distinct patterns of dysgraphia correspond to different neuroanatomical or inflammatory mechanisms within ICANS.

### 3.8 Evaluation challenges in writing disabilities related to immunotherapy

In addition to clinical characterization, several challenges remain in the objective evaluation of writing impairment during immunotherapy. The ICE grading system includes a sentence-writing task in which patients are asked to write a simple sentence. However, the scoring primarily evaluates whether a sentence can be produced and does not capture qualitative abnormalities in handwriting. Recent immunotherapy-focused reviews have also noted limitations in capturing higher-order cognitive symptoms, including writing abnormalities, within current evaluation frameworks (33). We experienced cases in which patients receiving T-cell engager therapies developed varying degrees of handwriting impairment depending on the type of letter (1); however, we were unable to assess the severity of the handwriting impairment. Therefore, we reviewed available reports on handwriting impairment during immunotherapy, current assessment approaches, and the potential application of artificial intelligence (AI) to handwriting assessment.

## 4 Discussion

Despite increasing recognition of ICANS as a clinically significant neurotoxicity, dysgraphia remains insufficiently characterized, under-recognized, and rarely assessed as an independent clinical feature. Recent reviews have begun to highlight dysgraphia as a potentially important but under-characterized manifestation of immunotherapy-associated neurotoxicity (34). In most clinical reports, dysgraphia is embedded within broader cognitive or language disturbances and is not systematically assessed. This gap between clinical relevance and limited structured evaluation highlights the need for a focused synthesis of ICANS-associated dysgraphia. Accordingly, this review aims to clarify its clinical characteristics, underlying mechanisms, and limitations of current assessment frameworks. This limitation may reflect the limited number of studies examining dysgraphia and the lack of structured characterization of writing abnormalities in immunotherapy (35, 36), as well as the limited number of reports examining chemotherapy and dysgraphia (37). It may also be related to the infrequent reports describing qualitative characteristics of letters among patients with dysgraphia during immunotherapy (2, 10, 38), possibly because it remains uncertain whether differences in disability are associated with differences in letter characteristics (1). The current review highlights the need for more structured approaches to evaluate the severity of dysgraphia.

### 4.1 Could relevant studies have been missed due to the narrow focus on writing impairment?

To minimize the risk of missing relevant studies, we intentionally expanded the search terms to include aphasia, language disturbance, and cognitive dysfunction, which frequently encompass writing impairment in clinical practice. Furthermore, studies were included even when writing impairment was implicitly evaluated as part of standardized neurotoxicity grading systems, such as the ICE score used in ICANS assessment. Notably, the inclusion of Fontanelli et al. (8) through manual screening highlights that clinically relevant writing abnormalities may be embedded within broader descriptions of frontal dysfunction and may not be captured by keyword-based searches alone.

### 4.2 Why were large clinical trials of CAR T-cell or T-cell engager therapy excluded?

Large clinical trials primarily focus on efficacy and global safety outcomes and often report neurotoxicity as aggregate categories without detailing specific higher-order cognitive symptoms, including writing impairment. For example, pediatric CAR-T studies have reported neurotoxicity primarily in terms of language and cognitive dysfunction without specific evaluation of writing impairment (38). As the aim of this review was to characterize writing impairment specifically, such trials were excluded unless they provided explicit or evaluative information relevant to writing or higher-order cognitive dysfunction.

### 4.3 Why does immunotherapy cause writing disorders?

Writing disorders therefore reflect dysfunction of distributed cortical networks involved in language, visuospatial processing, and motor planning (5, 6). Compared with immune effector cell therapies, classical chemotherapeutic agents appear less frequently associated with higher-order writing disturbances (35–37). In contrast, the emergence of immunotherapies such as CAR-T cells and T-cell engagers has been accompanied by reports of dysgraphia in the context of ICANS. Although dysgraphia exhibits heterogeneous patterns (**Figure 4**), current assessment systems such as the ICE grading system evaluate only the presence or absence of writing ability and do not capture qualitative abnormalities. Several reports have described dysgraphia in patients receiving immunotherapy, typically characterized by reduced legibility, distortion of letter forms, and perseverative writing errors (2, 4, 10, 19). In our previous study, handwriting abnormalities were more readily detectable when complex characters were used (1). In contrast, this review focuses specifically on dysgraphia induced by immunotherapy-associated ICANS, which is typically transient and associated with reversible cortical dysfunction (1, 7). Although the precise mechanisms of ICANS remain incompletely understood, current evidence suggests that cytokine-mediated neuroinflammation and blood–brain barrier disruption play central roles (25–28). Clinically, ICANS may present with encephalopathy, language disturbance, seizures, and early writing impairment (**Figure 5**) (9, 15). While ICANS monitoring relies on neuroimaging and the ICE grading system, the writing component of ICE is evaluated in a binary manner and does not assess qualitative features such as repetition, spatial distortion, or motor planning deficits. These findings suggest that dysgraphia in ICANS is not merely a secondary symptom but may reflect network-level cortical dysfunction.

### 4.4 Progress in developing the ICE grading system

The association between CAR-T and CNS toxicity has been evident since the earliest clinical trials on CAR-T therapy (12–14). The clinical manifestations of CNS toxicity include headaches, dizziness, delirium, seizures, aphasia, hallucinations, and temporary loss of motor and language skills. At the time, the CTCAE included seizures, encephalopathy, tremors, delirium, and a limited number of other neurological symptoms; however, it was not specifically designed for CAR-T cell therapy and did not allow for the assessment of the nature or severity of all neurological symptoms. To prospectively assess neurotoxicity, a phase I trial on anti-CD22 CAR-T therapy assessed four domains (attention, working memory, cognitive flexibility, and processing speed) in addition to baseline brain magnetic resonance imaging (39). Moreover, a CARTOX working group of experts was formed to develop a clinical severity rating chart for CNS neurotoxicity, a new neurotoxicity that emerged after CAR-T therapy administration, which prompted the creation of a new CNS toxicity assessment scale (CARTOX10) (10).

**Figure 7:**
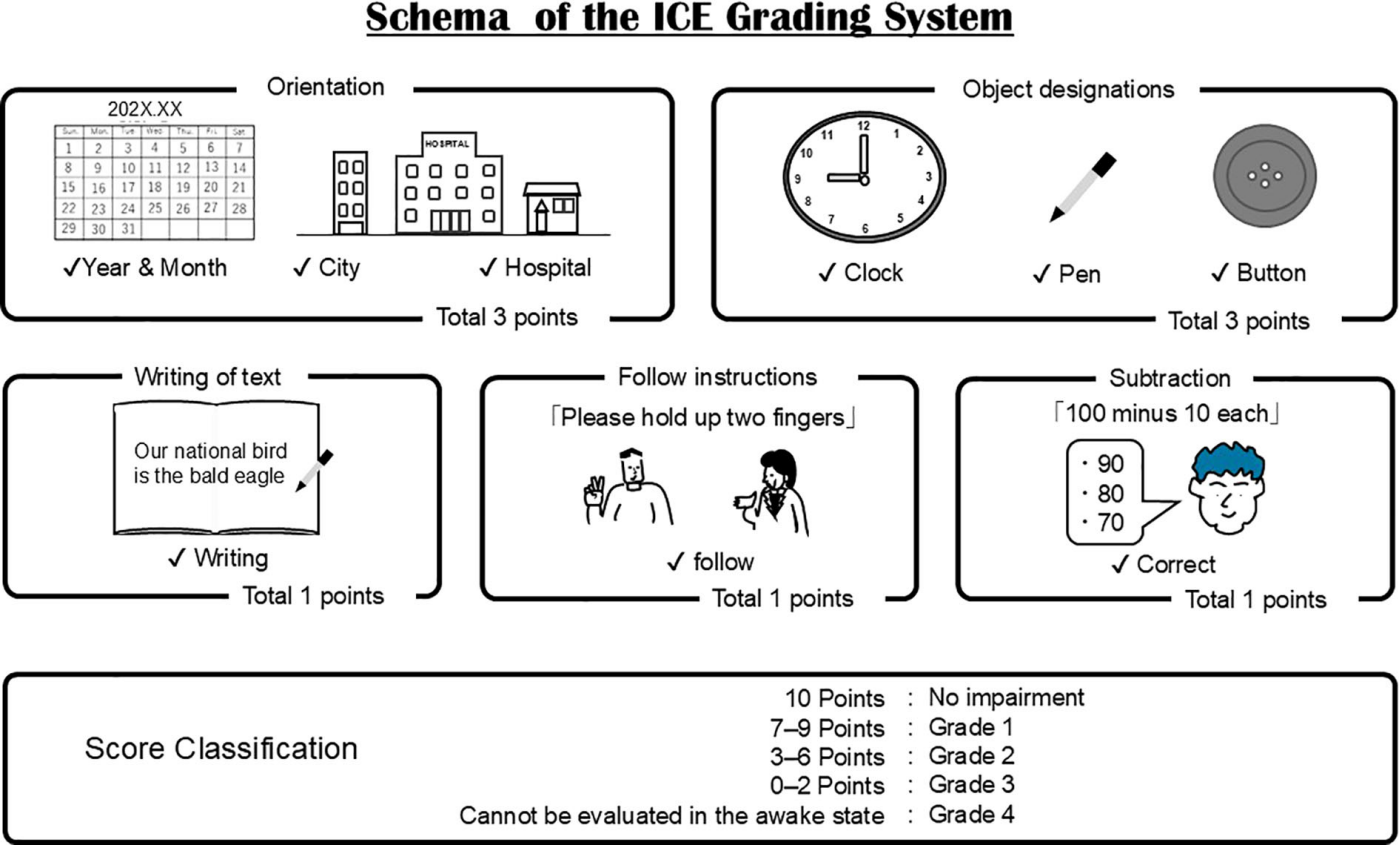
Schematic overview of the ICE grading system. The ICE score is a 10-point assessment tool used to evaluate neurotoxicity in patients receiving immunotherapy. It includes orientation, naming, writing, following commands, and subtraction tasks. The writing component assesses the ability to produce a sentence but does not evaluate qualitative handwriting abnormalities.

CARTOX10 is a 10-point scale that assesses orientation, naming, following commands, writing, and attention. This scale represents one of the first structured attempts to incorporate a writing task into neurotoxicity monitoring in cancer therapy. However, one drawback of CARTOX10 is that it cannot be readily applied to children under the age of 12 years, as they may not have sufficient cognitive ability to complete the assessment. Around this period, CNS toxicity emerged as an important adverse event associated with CAR-T therapy and T-cell engagers such as blinatumomab (16, 33). Subsequently, with support from the American Society for Transplantation and Cellular Therapy, standardized guidelines for cytokine release syndrome and neurotoxicity were developed. In this process, the CARTOX10 assessment was refined and simplified, leading to the development of the ICE score, which retains key elements such as orientation, naming, following commands, writing, and attention in a more standardized format (9) (**Figure 7**). Considering that the clinical trials leading up to the launch of tisagenlecleucel had unclear monitoring of neurological events, CARTOX and ICE were used to regrade neurological events. To monitor neurotoxicity among patients below 12 years of age, researchers decided to use the Cornell Assessment of Pediatric Delirium for delirium assessment (40), which is considered a highly sensitive screening tool for ICANS (41) and can be used from age 0. However, this tool contains no items that assess writing (42).

To summarize the evolution of neurotoxicity assessment in CAR-T therapy, prospective data collection from early clinical studies incorporated multiple neurocognitive parameters, including writing performance. These efforts contributed to increasing recognition of dysgraphia as a clinically relevant manifestation of immunotherapy-associated neurotoxicity. Subsequently, the ICE grading system was developed as a refined and standardized tool, incorporating a sentence-writing task (“Write a sentence”) as part of its assessment. However, despite the inclusion of this task, no structured components were introduced to evaluate the qualitative features or severity of writing impairment, potentially leading to under-recognition in clinical practice.

### 4.5 Dysgraphia in immunotherapy: clinical observations

Since the incorporation of the ICE grading system into ICANS monitoring, dysgraphia has become increasingly recognized in patients receiving immunotherapy (16, 19–21). Several reports have described dysgraphia in this context, typically characterized by reduced legibility, distortion of letter forms, and perseverative writing errors. In a representative study combining a writing task with the Mini-Mental State Examination (MMSE), handwriting became severely distorted following the onset of ICANS, accompanied by a mild decline in MMSE scores (10, 11). Other reports have described difficulty in writing or reading letters at the onset of neurotoxicity (19), progression to repetitive writing patterns with increasing ICANS severity (4), and early repetition of letters followed by more pronounced dysgraphia as neurotoxicity worsened (2). In our previous study, handwriting abnormalities during blinatumomab therapy were more readily detectable when complex characters were used, and were characterized by imbalance, blurred strokes, and impaired line control (1). Across these reports, dysgraphia typically appears in the early phase of ICANS and is often reversible, suggesting transient dysfunction of cortical networks rather than permanent structural damage. Despite the relatively high prevalence of ICANS, only a limited number of dysgraphia cases have been described (22, 23), likely reflecting under-recognition and the absence of structured evaluation methods. Current assessment systems primarily evaluate the presence or absence of writing ability and provide limited information on qualitative features such as distortion, repetition, or motor planning deficits. In addition, the degree and presentation of dysgraphia may vary depending on the type and complexity of writing tasks. The CTCAE version 5.0 includes only a grade 3 description related to severe communication impairment, without specific criteria for grading dysgraphia. Similarly, existing descriptions of the ICE grading system indicate that writing is assessed in a largely binary manner (i.e., whether the patient can write a sentence), without evaluation of qualitative abnormalities. These limitations highlight the lack of a standardized grading system for dysgraphia. Taken together, these findings suggest that dysgraphia associated with ICANS represents a heterogeneous clinical manifestation of network-level cortical dysfunction rather than a direct neurotoxic effect comparable to that observed with conventional chemotherapy.

### 4.6 Differences between immunotherapy and classical chemotherapy

Researchers have long sought to determine why classical chemotherapy is less likely to cause dysgraphia. From a functional perspective, neurological complications of chemotherapy can be broadly classified into central nervous system (CNS) injury and peripheral neuropathy, each arising through distinct mechanisms.

Central nervous system injury, including leukoencephalopathy affecting the myelin sheath of cerebral white matter, has been reported with antimetabolites, cytotoxic anticancer drugs, and taxanes (43–45). Hinchey et al. (46) first described reversible posterior occipital lobe leukoencephalopathy syndrome (PRES) in 1996, which is primarily associated with vasogenic edema, and subsequent studies have reported PRES following treatment with molecular-targeted agents and antibody therapies (47–53). CNS complications remain relatively uncommon because the blood–brain barrier restricts the penetration of many anticancer agents into the brain (54–57). In addition to regulating the exchange of substances between blood and brain tissue, the blood–brain barrier protects the CNS from toxic substances and pathogens, thereby maintaining normal brain function (58–60). Because dysgraphia generally requires disruption of brain parenchymal function, reports of dysgraphia associated with chemotherapy are limited, partly due to the rarity of CNS involvement and the lack of routine monitoring for writing disturbances (35, 36).

Chemotherapy-induced peripheral neuropathy (CIPN) has been reported following treatment with platinum compounds, taxanes, vinca alkaloids, and proteasome inhibitors (61–64). Unlike the brain, peripheral nerves lack a protective structure equivalent to the blood–brain barrier and are therefore more susceptible to direct drug-induced injury (65, 66). Neuropathy may involve axonal damage, neuronal cell bodies, or myelin and can persist for prolonged periods once symptoms develop (67, 68). Importantly, the pathophysiology and clinical manifestations of CIPN differ from those of ICANS. One of the few studies investigating handwriting in the context of CIPN was conducted by Hartman et al. (37) using the BHK scale, a standardized assessment tool for children’s handwriting. That study evaluated writing quality and speed in children at least one year after completion of vincristine-containing regimens for hematologic malignancies and found no long-term impairment in handwriting performance (37). Although “difficulty writing” may occasionally be reported in CIPN and appears in CTCAE descriptions, CIPN primarily reflects peripheral nerve dysfunction rather than a writing disorder caused by central brain dysfunction.

Consistent with these differences, only a limited number of reports describe dysgraphia during conventional chemotherapy, which may explain why no dedicated grading system for dysgraphia exists in the CTCAE.

### 4.7 Various methods for evaluating written text

Since Thorndike’s first writing test (69) in the early 20th century, many writing tests have been developed to evaluate handwriting quality (70–72). Numerous countries worldwide have created and continue to develop writing tests (73–79). Handwriting products are evaluated based on various characteristics, including letter size and shape, spatial organization of handwriting on paper, and margins. Handwriting evaluation is critical as it reveals information about an individual’s neuromotor and cognitive status.

Most of these assessment methods involve writing letters within a set time limit according to specific criteria, after which an expert evaluates the writing based on these criteria and calculates the subscores. The majority of evaluation criteria include “speed” and “legibility.” For example, the BHK test is conducted using paper and pen (80, 81). This test is a rapid assessment scale for evaluating children’s writing abilities and is considered to be the gold standard for diagnosing dysgraphia in France. In this test, children copy a passage from simple monosyllabic to more complex words onto a paper within 5 min. The evaluation is conducted based on two aspects: First is the writing speed. The number of characters written within 5 min is determined. Second is the writing quality. The first five sentences of the passage are evaluated semi-quantitatively on 13 items (e.g., shape, size, placement, and spacing of characters), generating a writing quality score. This score is a degradation score, indicating more errors and lower quality with higher scores.

The BHK test is conducted based on the complexity of words, not the complexity of characters, which may yield different outcomes. In other words, even if the BHK test includes many simple characters, it results in complex words. The fact that we did not find disabilities in simple characters but did in complex characters suggests that the BHK test is insufficient.

Furthermore, scoring some parameters relies on human judgment, which may introduce bias and take time to obtain results (82).

In handwriting science, subjective evaluation by humans is the mainstay. However, advancements in electrical and electronic engineering have now made it possible to draw letters on a tablet and have them evaluated by a computer or artificial intelligence (83). These new technologies have been applied in the evaluation of writing, allowing researchers to determine not only the written characters but also the angle and length of the lines, the speed and pressure of pen movement during writing, the movement of the pen in the air, and the balance and view of the characters, which cannot be determined by the human eye alone (82, 84–86). This new technology has also been applied to chemotherapy. In a 2001 report, a tablet and a force-sensing pen were used to investigate handwriting dexterity in children undergoing chemotherapy for acute lymphoblastic leukemia by having them draw at varying degrees of complexity (87). Thus, the use of electronic devices may be an objective and useful approach for assessing handwriting analysis (82, 83, 88–90). Moreover, in recent years, deep learning techniques, such as convolutional neural network (CNN), have been used to analyze letters (88, 89)- (91). New sensor-based tools have also been developed over the past few years (88, 90). We have found no reports of these advanced devices being used for writing impairment in cancer therapy.

### 4.8 Future directions for the assessment of dysgraphia in cancer immunotherapy

The CTCAE framework has not yet established a structured method for evaluating dysgraphia. Because dysgraphia must be assessed during treatment, practical and rapid approaches are needed. Considering that immunotherapy is administered to both children and adults, evaluation methods based on simple letters or figures that even preschool children can produce may be desirable.

However, our group also found that the appearance of dysgraphia may differ depending on the letters being evaluated (1); therefore, the complexity of the written characters should also be considered. In this context, future incorporation of dysgraphia into broader adverse-event assessment frameworks, such as the CTCAE, may be worth considering. Emerging evaluation approaches using artificial intelligence (AI), which has rapidly developed in recent years, may also provide complementary tools for the objective analysis of handwriting abnormalities.

At present, no standardized evaluation method exists for dysgraphia during cancer therapy, and differences in assessment across institutions may hinder accurate clinical interpretation. Prospective studies may therefore be required to establish practical and reproducible assessment strategies.

Future research should involve multidisciplinary collaboration among neurologists, hematologists, neuropsychologists, and clinical pharmacologists to better characterize the spectrum of ICANS-associated dysgraphia. Beyond its role as a clinical observation, dysgraphia may serve as a potential bedside indicator of higher-order cortical dysfunction during immunotherapy. Because writing integrates language processing, executive control, visuospatial organization, and motor planning, subtle disturbances in handwriting may reflect early disruption of distributed cortical networks before more severe neurological symptoms become evident.

In this context, structured handwriting assessment could complement existing neurotoxicity monitoring tools and provide a sensitive indicator of evolving ICANS. Future prospective studies integrating standardized handwriting tests, neuroimaging, and inflammatory biomarkers may further clarify the relationship between dysgraphia and the underlying neurobiological mechanisms of ICANS. The available evidence suggests that dysgraphia observed during ICANS represents a heterogeneous spectrum of writing abnormalities rather than a single uniform phenomenon (1–3, 8).

## 5 Conclusions

Considering our clinical observations that the degree of dysgraphia may vary depending on the complexity of written characters, there is a clear need to establish evaluation criteria that incorporate more detailed assessment of writing performance. Despite scattered reports on immunotherapy-associated dysgraphia, only a limited number of studies have systematically examined this phenomenon, highlighting a significant gap in current neurotoxicity assessment frameworks. Importantly, this narrative review does not propose a validated diagnostic algorithm or an immediately applicable solution. Rather, it aims to clarify the current limitations in the assessment of writing impairment during immunotherapy and to define unmet clinical needs in this emerging field. By positioning dysgraphia as a potentially sensitive clinical marker (1–4) of higher-order cognitive dysfunction within ICANS, this review underscores the importance of structured writing assessment in routine clinical practice. Furthermore, while artificial intelligence–based approaches are discussed as a future perspective, their role is conceptual at this stage and depends on the accumulation of standardized, high-quality handwriting data. Future research should focus on the development and validation of feasible, reproducible writing assessment tools before advanced analytical methods can be meaningfully applied.

## Supplementary material

Supplementary Table 1 Search strategy and results.

Supplementary Table 2 Full-text assessed studies and reasons for exclusion.

Supplementary Table 3 summarizes the studies assessed in full text and the reasons for exclusion. Studies were excluded if they did not explicitly describe writing impairment (e.g., dysgraphia, agraphia, or handwriting abnormalities) in the context of immunotherapy-related neurotoxicity.
