# Supplementary material for: Immunotherapy-associated dysgraphia as an early neurocognitive manifestation of immune effector cell–associated neurotoxicity syndrome: clinical characteristics, mechanistic insights, and assessment challenges
Source: Front Immunol. 2026 Apr 29;17:1731798. doi: 10.3389/fimmu.2026.1731798 (PMC13167948; doi:10.3389/fimmu.2026.1731798)
Supplement: Supplementary Table 1 — Search strategy and results. [file Presentation1.pptx]

## Slide 1
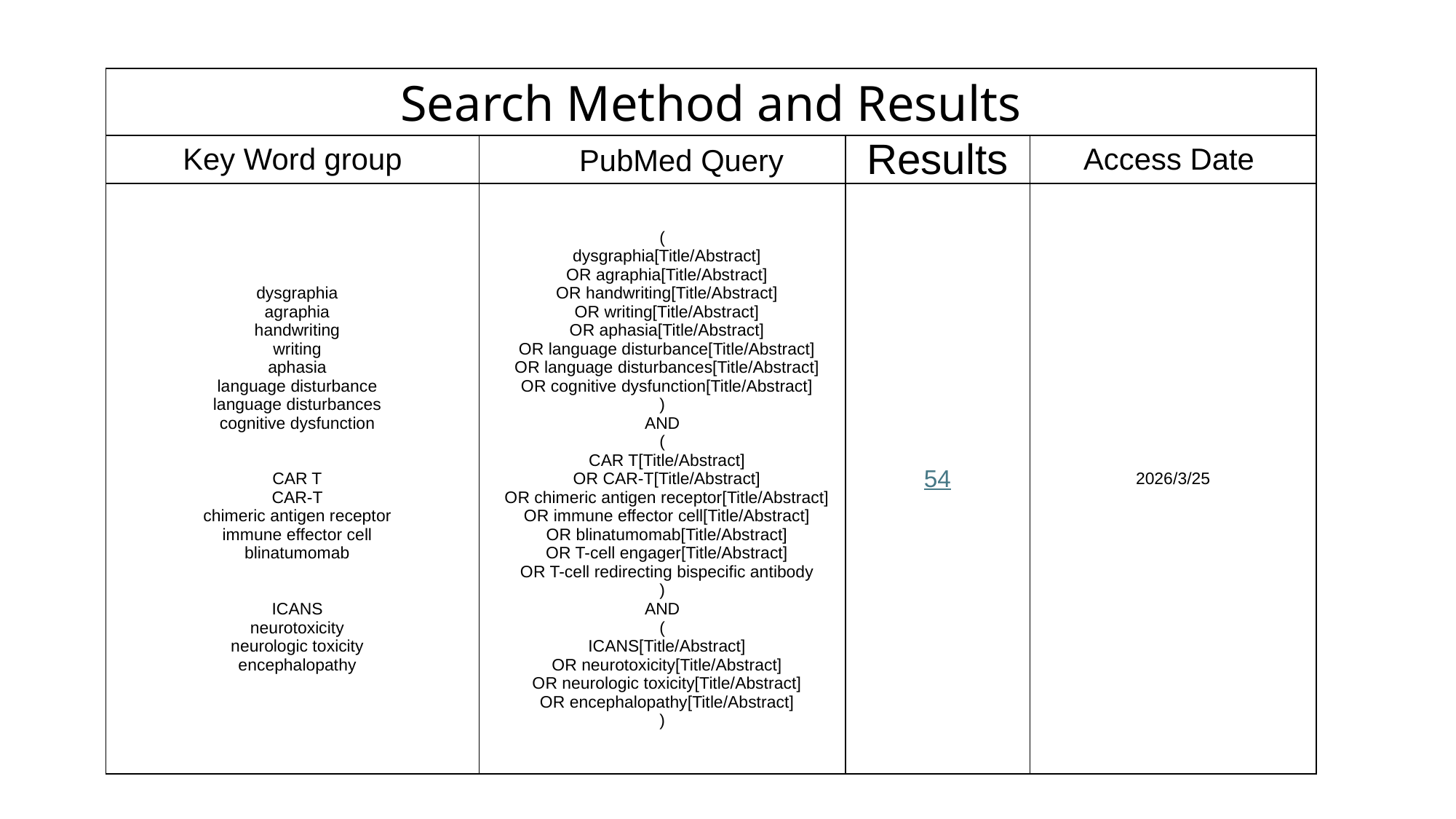

| Search Method and Results | | | |
| --- | --- | --- | --- |
| Key Word group | PubMed Query | Results | Access Date |
| dysgraphia agraphia handwriting writing aphasia language disturbance language disturbances cognitive dysfunction CAR T CAR-T chimeric antigen receptor immune effector cell blinatumomab ICANS neurotoxicity neurologic toxicity encephalopathy | ( dysgraphia[Title/Abstract] OR agraphia[Title/Abstract] OR handwriting[Title/Abstract] OR writing[Title/Abstract] OR aphasia[Title/Abstract] OR language disturbance[Title/Abstract] OR language disturbances[Title/Abstract] OR cognitive dysfunction[Title/Abstract] ) AND ( CAR T[Title/Abstract] OR CAR-T[Title/Abstract] OR chimeric antigen receptor[Title/Abstract] OR immune effector cell[Title/Abstract] OR blinatumomab[Title/Abstract] OR T-cell engager[Title/Abstract] OR T-cell redirecting bispecific antibody ) AND ( ICANS[Title/Abstract] OR neurotoxicity[Title/Abstract] OR neurologic toxicity[Title/Abstract] OR encephalopathy[Title/Abstract] ) | 54 | 2026/3/25 |
