# Supplementary material for: Immunotherapy-associated dysgraphia as an early neurocognitive manifestation of immune effector cell–associated neurotoxicity syndrome: clinical characteristics, mechanistic insights, and assessment challenges
Source: Front Immunol. 2026 Apr 29;17:1731798. doi: 10.3389/fimmu.2026.1731798 (PMC13167948; doi:10.3389/fimmu.2026.1731798)
Supplement: Supplementary Table 3 — summarizes the studies assessed in full text and the reasons for exclusion. Studies were excluded if they did not explicitly describe writing impairment (e.g., dysgraphia, agraphia, or handwriting abnormalities) in the context of immunotherapy-related neurotoxicity. [file Presentation2.pptx]

## Slide 1
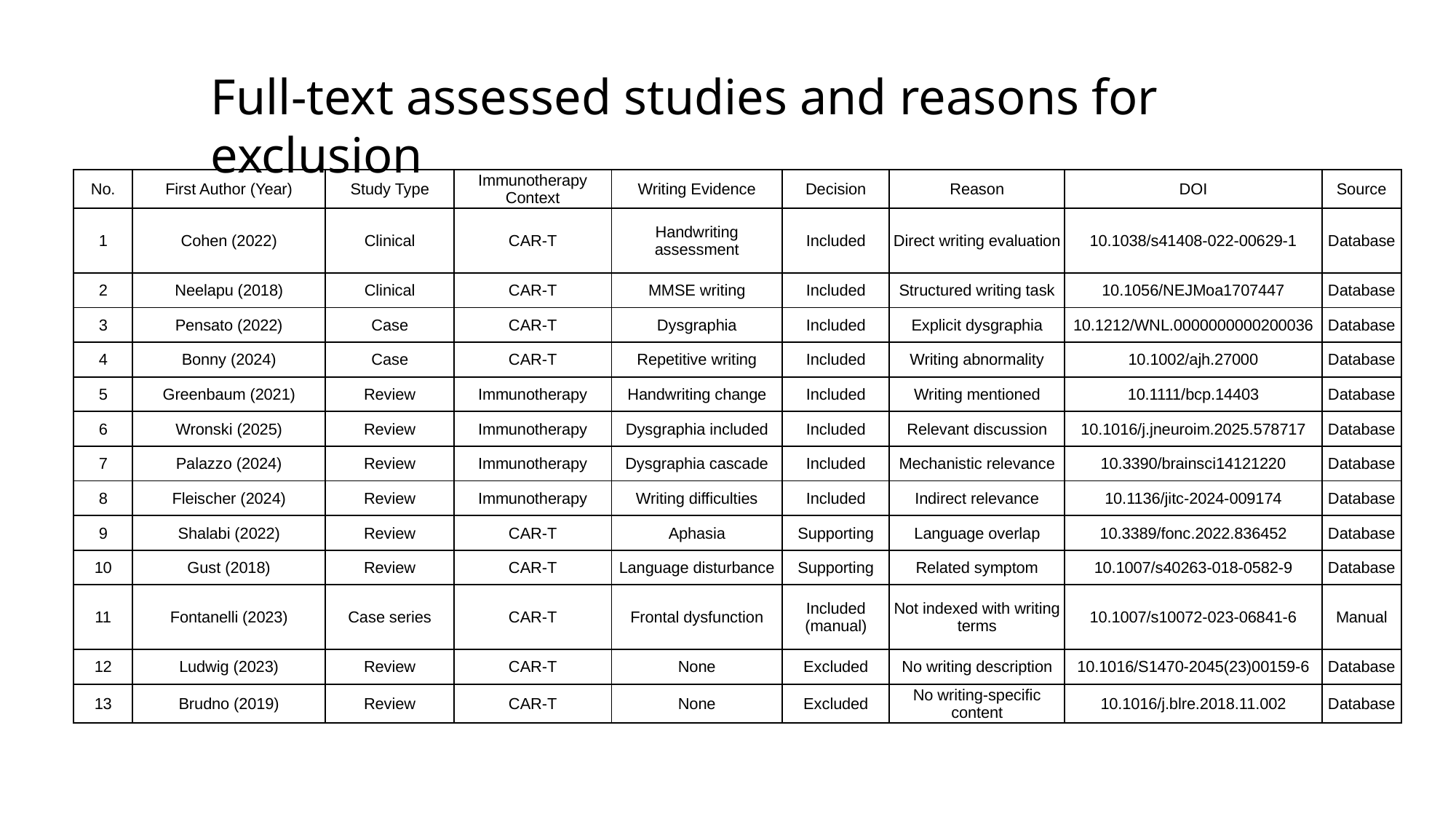

Full-text assessed studies and reasons for exclusion
| No. | First Author (Year) | Study Type | Immunotherapy Context | Writing Evidence | Decision | Reason | DOI | Source |
| --- | --- | --- | --- | --- | --- | --- | --- | --- |
| 1 | Cohen (2022) | Clinical | CAR-T | Handwriting assessment | Included | Direct writing evaluation | 10.1038/s41408-022-00629-1 | Database |
| 2 | Neelapu (2018) | Clinical | CAR-T | MMSE writing | Included | Structured writing task | 10.1056/NEJMoa1707447 | Database |
| 3 | Pensato (2022) | Case | CAR-T | Dysgraphia | Included | Explicit dysgraphia | 10.1212/WNL.0000000000200036 | Database |
| 4 | Bonny (2024) | Case | CAR-T | Repetitive writing | Included | Writing abnormality | 10.1002/ajh.27000 | Database |
| 5 | Greenbaum (2021) | Review | Immunotherapy | Handwriting change | Included | Writing mentioned | 10.1111/bcp.14403 | Database |
| 6 | Wronski (2025) | Review | Immunotherapy | Dysgraphia included | Included | Relevant discussion | 10.1016/j.jneuroim.2025.578717 | Database |
| 7 | Palazzo (2024) | Review | Immunotherapy | Dysgraphia cascade | Included | Mechanistic relevance | 10.3390/brainsci14121220 | Database |
| 8 | Fleischer (2024) | Review | Immunotherapy | Writing difficulties | Included | Indirect relevance | 10.1136/jitc-2024-009174 | Database |
| 9 | Shalabi (2022) | Review | CAR-T | Aphasia | Supporting | Language overlap | 10.3389/fonc.2022.836452 | Database |
| 10 | Gust (2018) | Review | CAR-T | Language disturbance | Supporting | Related symptom | 10.1007/s40263-018-0582-9 | Database |
| 11 | Fontanelli (2023) | Case series | CAR-T | Frontal dysfunction | Included (manual) | Not indexed with writing terms | 10.1007/s10072-023-06841-6 | Manual |
| 12 | Ludwig (2023) | Review | CAR-T | None | Excluded | No writing description | 10.1016/S1470-2045(23)00159-6 | Database |
| 13 | Brudno (2019) | Review | CAR-T | None | Excluded | No writing-specific content | 10.1016/j.blre.2018.11.002 | Database |
